# Supplementary material for: Are carbon emissions trading and green financial instruments synergistic? -Comprehensive quantitative research based on content analysis
Source: PLoS One. 2024 Mar 7;19(3):e0298601. doi: 10.1371/journal.pone.0298601 (PMC10919601; doi:10.1371/journal.pone.0298601)
Supplement: S1 Data — (ZIP) [file pone.0298601.s001.zip › Data/地级市Z_F工作报告.zip!/╘╞─╧/┴┘▓╫╩╨/└┤╫╘╣1⁄2╓┌║┼í╛┬φ┐╦╩2╛▌═°í┐_2017.docx]

各位代表：

　　现在，我代表市人民政府，向大会报告工作，请予审议，请政协委员和列席人员提出意见。

　　一、2016年工作回顾

　　2016年，是临沧大事多、好事多、喜事多的一年。在省委、省政府和市委的坚强领导下，在市人大及其常委会的法律监督、工作监督和市政协的民主监督下，市人民政府紧密团结在以习近平同志为核心的党中央周围，全面贯彻落实党的十八大和十八届三中、四中、五中、六中全会精神，深入学习贯彻习近平总书记系列重要讲话特别是考察云南重要讲话精神，认真贯彻刘云山等党和国家领导同志考察临沧重要指示精神，统筹推进“五位一体”总体布局和协调推进“四个全面”战略布局，落实新发展理念，立足于经济新常态下临沧发展的阶段性特征，主动服务和融入国家战略，按照市委三届八次全会和市第四次党代会的决策部署，全力稳增长、调结构、兴产业、惠民生、补短板，全市经济发展、民族团结、边境稳定、社会和谐，实现了“十三五”良好开局，支撑跨越式发展的“四梁八柱”已经落地，发展的大框架、大格局基本形成。

　　一年来，我们主要抓了以下八个方面工作：

　　（一）全力稳定经济增长，经济总量不断壮大

　　始终坚持发展第一要务，千方百计稳增长。“十三五”规划顺利实施。研究出台落实稳增长22个方面59条政策措施。认真抓好省、市“四个一百”重点项目，集中开工41个重点项目。筹集资金3.6亿元专项用于铁路、高速公路、重大水利工程等重点项目前期工作。地方政府新增债券转贷资金13.3亿元，存量置换债券转贷资金30.5亿元，争取到国家专项建设基金59.2亿元。扎实开展服务企业年活动，发放应急互助资金贷款1.2亿元，支持银行调头资金1.9亿元。全市经济稳中有进，实现生产总值552.4亿元，增长10.2%；完成规模以上固定资产投资917亿元，增长25.8%；地方财政总收入完成59.3亿元，增长1.9%，其中，一般公共预算收入38.3亿元，同口径增长2.6%；一般公共预算支出213.4亿元，增长5.4%；社会消费品零售总额173.7亿元，增长12.4%；外贸进出口总额45.9亿元，增长17%；城镇常住居民人均可支配收入23072元，增长8.7%；农村常住居民人均可支配收入8914元，增长10.5%；居民消费价格总水平累计涨幅1.6%。较好完成了市三届人大三次会议确定的目标任务。

　　（二）主动服务和融入国家战略，“五网”基础设施建设取得实质性突破

　　抢抓临沧被列为全省五出境通道之一的重大机遇，以高速公路、铁路为主的综合交通网络构架基本形成。大临铁路完成投资9.5亿元，临沧至清水河、临沧至普洱、临沧至芒市铁路进入国家中长期铁路网规划。墨临高速临沧境内段完成投资8.2亿元，机场高速完成投资12.3亿元。新开工建设临沧至清水河、临翔至双江、南伞至清水河高速公路，临清高速完成投资5.7亿元。云县至临翔、云县至凤庆高速公路“工可”获批。永德（链子桥）至耿马（勐简）、耿马（勐撒）至沧源（糯良）、南涧至云县、沧源（糯良）至岗莫标山高速公路前期工作加快推进。沧源机场建成通航，凤庆通用机场开工建设，临沧机场改扩建顺利推进，孟定支线机场、永德通用机场前期工作加快推进。在建的33件重点水利工程累计完成投资41亿元，耿马灌区加快建设，凤庆中心城区供水工程、4件小（一）型水库和2件连通工程开工建设，2件中型水库、5件小（一）型水库投入使用。500千伏博尚变电站建成使用。临翔、云县、凤庆天然气利用加快推进。宽带网络和4G网络实现乡镇全覆盖。

　　（三）着力建设辐射中心前沿窗口，对外开放水平不断提高

　　以对缅开放为重点，辐射中心前沿窗口建设取得新突破。清水河口岸边民互市贸易市场建成投入使用，清水河食用水生动物指定口岸建设顺利启动。中国清水河至缅甸果敢、滚弄、腊戍三条跨境旅游线路开通运营。临沧市驻缅甸腊戍、内比都商务代表处挂牌成立。国家援建缅甸滚弄大桥协议正式签订。口岸通关便利化水平不断提高，口岸基础设施建设稳步推进。与缅北地区合作建成特色产业基地114.5万亩，38户临沧企业在缅北地区投资发展。沿边金融综合改革试验区建设稳步推进。边合区管理体制机制进一步完善，承接产业转移取得新进展，摩托车组装、果蔬包装等一批外向型项目建成投产。全市招商引资市外实际到位资金达1009.9亿元，增长21.6%。积极参加南亚博览会等国际大型会展，成功举办第六届边境经济贸易交易会、第四届亚洲微电影艺术节等重大活动，开放、包容、发展的临沧新形象得到进一步展现。

　　（四）强力推进精准扶贫，民族团结进步更加巩固

　　坚持把脱贫攻坚作为第一民生工程来抓。全面实施“27241”脱贫攻坚工程，争取到易地扶贫搬迁专项贷款33.2亿元，启动建设易地扶贫搬迁三年行动计划集中安置点172个，开工建设农村危房改造和抗震安居工程8.4万户，竣工7.1万户，完成投资101亿元。深入开展“挂包帮”、“转走访”活动，驻村扶贫工作队覆盖全部贫困村。云县有望实现脱贫摘帽目标。全市14个贫困乡镇、80个贫困村脱贫出列，减少贫困人口6.54万人。民族团结进步示范区建设、兴边富民工程改善沿边群众生产生活条件三年行动计划有力推进，民族地区基础设施加快改善。启动实施新一轮扶持彝族支系俐侎人发展三年专项规划和直过民族脱贫攻坚战计划，积极推进边疆解“五难”工程，采取“六个共同”以及党建扶贫“双推进”机制，精准推进脱贫攻坚，先进经验先后在《人民日报》、中央电视台等重要媒体上推广，得到党中央、省委、省政府领导的充分肯定。随着脱贫攻坚的深入推进，全市民族团结进步的基础更加巩固。

　　（五）发挥资源优势，产业发展向“两型三化”迈进

　　加快转换生物资源优势为产业、市场、品牌优势。完成中低产田地改造25.8万亩，粮食总产量达105.2万吨。着力发展茶叶、核桃、澳洲坚果、蔗糖、咖啡、烤烟等产业，新增高原特色产业基地49万亩，累计建成2151万亩，第一产业实现增加值154.7亿元，增长5.9%。一批产业发展配套项目建成投产，2户企业晋升为国家级农业产业化重点龙头企业。成功举办2016中国临沧（凤庆）红茶节。农业生产经营体系不断创新，“互联网+现代农业”加快发展，一批主营特色农产品平台投入运营。加快建设3个百亿元工业园区和县域工业园区，入园企业达408户。新增规模以上工业企业18户。实现规模以上工业增加值91.9亿元，增长12.3%；第二产业实现增加值187.3亿元，增长13.3%。生态文化旅游产业加速发展，沧源国际旅游度假区建设持续推进，旅游产业三年行动计划重点项目建设加快实施，“世界佤乡好地方·避暑避寒到临沧”的旅游品牌不断提升。全年接待海内外游客1291.9万人次，增长58.1%，实现旅游总收入112.1亿元，增长70.8%。实施省级电子商务兴边富民三年行动计划、全国电子商务进农村综合示范县建设项目，市级电子商务服务中心及7个县区的县级服务中心投入使用。第三产业实现增加值210.4亿元，增长10.6%。

　　（六）统筹城乡区域协调发展，生态文明建设走在前列

　　按照“一轴两城三带”的发展空间布局，发挥大临铁路、墨临高速的主轴带动作用，规划建设临沧城东片区，临沧中心城市扩容提质加快推进。按照岸城一体化要求，孟定新兴口岸城市规划建设有序推进。城市地下综合管廊和海绵城市建设前期工作扎实开展，城乡公厕、垃圾收运处理设施、城市污水管网建设加快推进，城镇基础设施公共服务体系不断完善，城镇化率达38.96%。启动耿马自治县国家中小城市综合改革试点建设。沧源自治县、双江自治县获得省级园林县城称号。户籍制度改革加快推进，城乡公共服务均等化水平不断提高。城镇保障性住房建设及棚户区改造三年行动计划取得新成效，城镇棚户区改造开工率100%。住房公积金制度覆盖率、信贷资金投放量不断加大。乡镇规划建设管理三年行动扎实推进，一批“小而美、小而干净、小而宜居”的特色城镇建设初显成效。农村宅基地和集体建设用地确权登记发证工作快速推进。城乡“四治三改一拆一增”和农村“七改三清”环境整治行动加快实施，“洁净临沧”行动取得良好成效，完成旧村改造1333个，美丽宜居乡村建设400个，洁净村庄创建845个，洁净庭院示范家园创建1万户。严格落实生态环境保护制度，推进退耕还林还草、荒漠化石漠化治理、重点区域生态修复、绿色银行建设，完成造林81.8万亩。南汀河沿线、澜沧江沿线、沿边一线生态综合治理深入实施，全国低碳国土实验区建设加快，生态建设与地质灾害防治相结合工作扎实推进，完成水土流失治理355平方公里。全面推进节能减排，完成省下达节能减排任务。深入实施水、大气等污染防治行动计划，县级及以上集中式饮用水水源地水质全面达标，主要城镇环境空气质量总体保持良好。

　　（七）加快改革创新步伐，发展内生活力进一步释放

　　以供给侧结构性改革为主线的各重点领域改革扎实推进，“三去一降一补”工作取得初步成效。涉及8大领域的26项年度重大改革事项稳步推进。完成党政机关公务用车制度改革。国有资产监管机制进一步完善。财税体制改革不断深化，“营改增”全面实施，严格控制和防范系统性金融风险。积极深化电力、水利、油、运等资源交易价格改革，取消矿产资源补偿费，取缔不合理的收费基金项目，减税3.5亿元，降费0.5亿元，降低用电成本0.6亿元。深化收入分配制度改革，推行企业工资集体协商制度，完善国有企业负责人薪酬制度，落实最低工资标准，健全工资增长机制，多渠道增加城乡居民收入，城乡及行业之间的收入差距逐步缩小。土地承包经营权确权登记颁证等农村改革有序推进，农垦、供销社、国有林场改革全面开展。全面完成商事登记制度改革。医药卫生体制改革不断深化，县级公立医院改革实现全覆盖。科技体制机制改革不断深化，企业创新主体地位不断提升，科技创新对经济增长的支撑引领作用不断增强。

　　（八）着力保障和改善民生，社会保持和谐稳定

　　10件惠民实事全面落实。各级各类教育协调发展，启动实施边境县14年免费教育，学前三年毛入园率达57.8%，义务教育巩固率达92%，高考一本上线率居全省第4位，现代职业教育体系加快构建，高等教育、特殊教育稳步发展。市县乡村医疗基础设施不断改善，重大疾病防控、妇幼保健和计划生育事业深入推进，全面两孩政策平稳有序实施。公共文化体系进一步构建，广播电视覆盖率达99%，成功举办第九届全省农民运动会和2016年全省少数民族传统体育锦标赛。社会保障体系更加完善，被征地农民养老保障政策试点稳妥推进，城乡居民医疗保险制度加快整合，机关事业单位养老保险入轨运行，全民参保登记计划有效实施，各项社会保险参保达204.3万人次。养老服务和社会福利体系不断完善，社会救助保障兜底工作有序推进。“10·7”、“3·01”地震受灾群众全部搬入新居。高度重视大中专毕业生就业，发放创业扶持贷款4.3亿元，城镇新增就业2.5万人，城镇登记失业率3.75%。依法管理宗教事务，全市宗教和谐稳定。国防动员、民兵预备役、双拥等工作取得良好成效，连续三届被评为“全国双拥模范城”。深入实施智慧边境建设，“2·09”维稳后续工作扎实开展，边境和谐稳定。高度重视安全生产，安全生产形势稳定向好。法治临沧建设全面推进，成为全省第一批行使地方立法权的市。圆满完成第三轮禁毒防艾和“六五”普法工作，平安临沧建设取得显著成效。社会矛盾纠纷排查调处机制不断完善，一批多年积累的矛盾问题和信访积案得到妥善化解，群众合法权益得到有效维护。积极支持工会、共青团、妇联、科协、文联、社科联、工商联、残联、侨联、红十字会等人民团体开展工作，气象、水文、地震、档案、地方志等工作不断加强，关工委、妇儿工委、老龄、慈善等事业不断巩固提升。

　　一年来，市人民政府始终高度重视自身建设。严格依法行政，政府职能加快转变，政府效能明显提高。“放管服”改革不断深化。市县区对应取消非行政许可项目共计205项，终结非行政许可事项的审批历史。电子政务建设步伐加快，有效降低交易成本。稳妥推进政府向社会购买服务。组建临沧仲裁委员会，推行政府法律顾问制度。市县乡政府权力清单和责任清单全面公布，“双随机、一公开”监管方式全面推开，行政权力运行更加规范。完善财政资金管理、公共资源交易制度建设，财政预决算、“三公经费”、重大项目实施、社会公益事业建设等领域的政务公开力度不断加大。自觉接受市人大及其常委会的法律监督、工作监督和市政协的民主监督，认真听取人大代表、政协委员、民主党派、工商联、无党派人士和各人民团体的意见，扎实办理人大代表建议和政协委员提案。加强工作绩效考核，完善奖惩机制。党风廉政建设和反腐败工作全面加强。加强行政监督问责，严格落实主体责任和监督责任，坚决整治不作为、乱作为等行为。“两学一做”学习教育扎实推进，干部能力素质进一步提高，想干事、能干事、干成事的导向鲜明，为全市经济社会发展注入了强大活力。

　　各位代表！2016年取得的成绩，是广大干部群众紧密团结在以习近平同志为核心的党中央周围，主动服务和融入国家战略的结果，是省委、省政府大力关心支持的结果，是市委正确领导、科学决策的结果，是市人大及其常委会和市政协加强监督、大力支持的结果，是全市各族人民团结拼搏、苦干实干的结果。在此，我谨代表市人民政府，向广大工人、农民、干部、知识分子，向人大代表、政协委员、各民主党派、工商联和无党派人士、各人民团体、社会各界人士，向驻临人民解放军和武警部队官兵，向所有关心支持临沧发展的港澳同胞、台湾同胞、海外侨胞和国际友好人士，表示崇高的敬意和衷心的感谢！

　　在取得成绩的同时，我们也清醒地认识到，在同步全面建成小康社会的道路上，我们仍然面临诸多困难和问题。经济量小质弱，同步建成小康社会的压力较大。供给侧结构性改革任务艰巨，经济转型发展的结构性矛盾突出。高原特色农业发展处在粗放型发展阶段，以市场为导向的产业创新能力仍然较低。工业经济困难问题突出，实体经济发展步履艰难。推进项目的机制、融资、人才等要素保障能力需要进一步加强。“2·09”维稳后续工作及维护边境和谐稳定的任务较重。部分干部主动服务和融入国家战略的意识不强，落实新发展理念的能力素质不能完全适应发展的需要。针对这些困难和问题，我们必须采取更有针对性的措施，确保问题得到解决。

　　二、2017年工作安排

　　2017年是决战脱贫攻坚、推进跨越式发展的重要之年。时代已经把临沧推到了一个新的起点上，实现跨越式发展具有千载难逢的重大机遇。党的光辉照边疆，党和国家领导人关心关注临沧，省委、省政府把临沧的发展放在更加突出的位置，为临沧发展把准了脉络、指明了方向，全市经济社会发展具有良好宽松的外部环境；国家“一带一路”倡议和云南辐射中心建设战略叠加临沧，铁路、高速公路、机场等重大基础设施项目快速推进，临沧正由开放末梢变为前沿；中缅两国交流合作不断深化，为临沧发挥无可替代的区位优势，多层次、宽领域推进对缅开放提供了良好的国际环境；全省“做强滇中、搞活沿边、联动廊带、多点支撑、双向开放”的区域协调发展新格局，为临沧深度融入全省发展大局搭建了良好平台；经过历届党委、政府和各族人民的艰苦奋斗，综合实力显著提升，发展基础更加厚实，为临沧实现跨越式发展打下良好基础。更为可贵的是，边疆人民心向党，临沧各族人民听党话、报党恩，具有思发展、谋发展的强烈愿望，凝聚了全面奔向小康社会的强大动力，我们有基础、有条件创“临沧速度”，实现跨越式发展。

　　今年政府工作的总体要求是：紧密团结在以习近平同志为核心的党中央周围，全面贯彻落实党的十八大和十八届三中、四中、五中、六中全会精神，以邓小平理论、“三个代表”重要思想、科学发展观为指导，深入贯彻习近平总书记系列重要讲话精神，统筹推进“五位一体”总体布局和协调推进“四个全面”战略布局，对标习近平总书记对云南的“三个定位”，落实中央经济工作会议、省第十次党代会、市第四次党代会和市委四届二次全会精神，贯彻新发展理念，坚持稳中求进工作总基调，以推进供给侧结构性改革为主线，以实现跨越式发展为目标，更加积极主动地服务和融入国家“一带一路”倡议和云南省面向南亚东南亚辐射中心建设，坚决打好工业经济攻坚战，纵深推进县域经济、园区经济、民营经济“三大战役”，做好稳增长、促改革、调结构、惠民生、防风险，突出抓好基础设施建设、产业转型升级、脱贫攻坚拔寨、沿边开发开放、生态文明建设、民生改善保障、维护边境稳定工作，鼓足干劲、奋勇争先，确保经济社会发展各项指标高开高走，为与全省全国同步全面建成小康社会打下坚实基础。

　　今年，经济社会发展的主要预期目标建议为：

　　——地区生产总值增长10%以上；

　　——规模以上固定资产投资增长25%；

　　——规模以上工业增加值增长12%；

　　—— 一般公共预算收入增长4%；

　　—— 一般公共预算支出增长5%；

　　——社会消费品零售总额增长13%；

　　——外贸进出口总额增长15%；

　　——城镇常住居民人均可支配收入增长9%；

　　——农村常住居民人均可支配收入增长10%；

　　——招商引资实际到位资金增长15%；

　　——城镇登记失业率控制在4.5%以内；

　　——居民消费价格总水平累计涨幅控制在3%左右；

　　——单位生产总值能耗降幅1.2%。

　　围绕上述目标，市人民政府将全力抓好以下八个方面的工作：

　　（一）积极推进供给侧结构性改革，加快实现新旧动能转换

　　坚持供需两端发力、市场主导和政府引导结合，着力在“降成本、补短板”上下功夫，有效落实“三去一降一补”任务。杜绝重复建设和低端产品开发，禁止新上过剩产能项目。去库存与促进常住人口市民化、完善住房保障体系有效结合，商品房去库存25%。尽快建立宏观金融战略平台，建立以财政为基本保障的还本付息机制和产业发展基金。强化银政企合作，实现新增融资50亿元以上。落实债转股政策，扩大直接融资。完成向上争取资金185亿元以上。进一步发挥保险支持经济社会发展作用。加大财源培植和税收征管力度，持续增加财政收入。用好用活地方债券发行政策，加强政府债务管理，防范金融风险和地方债务风险。继续扩大电力市场，完成市场化交易电量6亿千瓦时。全面推进“营改增”，落实国家减税降费政策，切实降低企业成本。千方百计补齐基础设施、产业发展、脱贫攻坚、科技创新、生态环保、社会事业等领域短板。

　　发挥投资对拉动经济增长的关键作用，保持投资较快增长。扎实抓好“四个一百”重点项目。强化要素保障，超常规、大力度服务保障在建新建项目推进。瞄准符合国家发展规划和政策投资导向，储备一批有利于全市补短板、打基础、强支撑的重大项目，引进并开工一批牵动力强的重大项目。做实项目前期工作，争取一批专项建设基金。确保规模以上固定资产投资完成1150亿元。

　　合理规划布局县域生产力，找准定位，扬长避短，在差异竞争中拓展发展空间，做强做大县域特色优势产业。支持县城提质扩容，提升产城融合，加快农业人口市民化。把县域经济发展与民营经济发展结合起来，充分挖掘民资、民智、民力，让民营经济成为壮大县域经济的重要支撑。严格县域经济考核，明确争先进位目标和举措，实现县域经济指标都有新突破。

　　（二）强力推进以铁路、高速公路为主的“五网”基础设施建设并实现新突破

　　加快推进大临铁路，完成投资13亿元，力争临清铁路上半年纳入国家勘察计划，下半年开工。全力推进“县县通高速公路”，完成高速公路投资110亿元。机场高速完成投资10亿元，实现年内基本建成；临清高速完成投资50亿元，实现孟定段30公里基本建成；墨临高速完成投资13.5亿元，争取临沧境内段全线开工；临双高速完成投资10亿元，重点推进博尚至肖家隧道、双江县城连接线工程建设；南清高速完成投资7亿元，重点推进南捧河枢纽、一碗水隧道、镇康县城连接线工程；云县至临翔、云县至凤庆高速2月底开工，完成投资14亿元；争取永德（链子桥）至耿马（勐简）、耿马（勐撒）至沧源（糯良）高速上半年开工，完成投资5.5亿元；争取南涧至云县、沧源（糯良）至岗莫标山高速年底前开工。积极推进小黑江至勐省、立新至孟定、龙镇桥至南伞、德党至勐堆国省干线公路建设，力争年内完成建设任务，完成投资4.4亿元。完成建制村通畅工程695公里，实现建制村公路100%通畅，启动直过民族、人口较少民族、边境自然村公路建设，完成农村公路投资18亿元。抓好临沧港前期工作，推进澜沧江244界碑至临沧港四级航道工程项目建设。加速推进孟定支线机场、永德通用机场前期工作，加快临沧机场改扩建和凤庆通用机场建设，完成民航投资1.3亿元。

　　开工建设耿马灌区主体工程、凤庆北部片区乡镇供水工程。争取开工4件中型水库，加快在建29件重点水源工程建设，力争竣工验收2件中型水库，2件小（一）型水库。推动3个小型农田水利重点县工程建设。力争有效灌溉保障率达50%，解决15万人农村饮水安全。积极推进对缅输电，加快500千伏孟定输变电工程前期工作。实施新一轮农村电网改造升级。加快城市天然气管网建设和城市天然气综合利用。强化通信基础设施建设，实现城市和农村光纤覆盖均达100%，4G网络100%覆盖。建设300个行政村电子商务点。着力发挥信息技术在新兴产业培育中的重要作用。

　　（三）推进产业转型升级，加快构建现代产业体系

　　坚持重农强农惠农不动摇，以实现“农业强、农村美、农民富”为目标，扎实抓好“三农”工作。积极推进农业供给侧结构性改革，优化蔗糖、茶叶、核桃、咖啡、橡胶、烤烟等农业产业结构，坚持市场导向，增加绿色优质农产品供给。实行最严格的耕地保护制度，完成中低产田地改造17.3万亩，稳定粮食生产。筹办好第十三届中国茶业经济年会。以打造“世界第一”为目标，建成国家坚果类检测重点实验室，加快申报临沧澳洲坚果国家农产品地理标志和地理标志证明商标，筹备好2018年第八届国际澳洲坚果研讨会。加快提升畜牧业、高原淡水养殖和休闲观光农业。加快培育新型职业农民，培育壮大新型经营主体和农业“小巨人”，新增农业产业化龙头企业10户以上，新发展农民专业合作社200个以上。发展村级集体经济，逐步消除“空壳村”。认真落实强农惠农政策，保障农民多渠道增收。开展农村土地“三权分置”改革，深化农垦、供销社、国有林场、农田水利等改革。扩大农业保险覆盖范围。全面完成第三次全国农业普查。

　　打响工业经济攻坚战。加快培育电矿结合载能产业、农产品精深加工产业以及对外加工贸易等产业。集中力量推动临沧工业园区、云县新材料光伏产业园区、凤庆滇红生态产业园区等重点产业园区发展，优化园区布局，明确主导产业定位，推进一批重点项目，打造专业化、特色化园区。实施上市公司减持股份扶持等政策，培育规模以上工业企业20户以上。继续深化商事制度改革，实施民营经济“双培双优”行动计划，实施“两个10万元”微型企业扶持900户以上，新增中小微企业2500户以上，民营经济增加值增长10%以上。推行工业经济效益通报制度。深化服务企业年活动，切实抓好企业解困工作，着力振兴实体经济。

　　提升传统服务业，促进转型升级。加快建设一批物流设施，促进现代物流业发展。加快融入全省服务经济“倍增”计划、“云上云”行动计划，推动现代金融、文化创意、电子商务等生产性服务业加快发展。加快实施生态文化旅游产业发展三年行动计划，创建临翔、沧源省级全域旅游示范区，以佤山机场通航为契机，把沧源国际旅游度假区作为临沧旅游发展的重要引擎，促进全市旅游产业上台阶，力争接待海外旅游者增长16%，接待国内旅游者增长32%，旅游业总收入增长46%。

　　（四）全力推进脱贫攻坚工程，加快贫困群众脱贫步伐

　　围绕“六个精准”，聚焦建档立卡对象特别是深度贫困人口，加快实施住房、健康、金融、旅游、基础设施等专项扶贫行动，改善农村贫困人口生产生活条件。持续推进易地扶贫搬迁工程和农村危旧房改造，完成危旧房改造7万户，克期完成临翔区、凤庆县、永德县、耿马自治县、双江自治县5个县脱贫摘帽，10个贫困乡镇、109个贫困村出列，净脱贫7.3万人以上目标。巩固提升云县脱贫攻坚成果，抓好迎检工作。加快沧源自治县、镇康县脱贫攻坚工作。

　　加强产业规划引导，健全产业到户到人精准扶持机制，大力发展特色产业。市级预算安排1亿元脱贫攻坚专项资金。加大贫困人口劳动力培训和转移力度，开展农村劳动力培训19万人次。继续推进“一村一名大学生”工程、“百企帮百校”和“1+1”城乡义务教育阶段学校结对帮扶工作。

　　严格落实各级党政“一把手”负总责的双组长扶贫开发工作责任制，实行最严格的年度脱贫攻坚报告、考核管理和督查审计制度，发挥社会监督作用，坚决防止数字脱贫、弄虚作假。加强农村贫困统计监测体系建设，提高监测能力和数据质量。继续深入开展“挂包帮”定点扶贫，不断强化责任落实与监督机制。主动对接“万企帮万村”精准扶贫行动，广泛动员驻临部队、企业、社会组织、公民参与扶贫。

　　（五）着力解决“五通”问题，提高沿边开发开放水平

　　坚持“四个先行”，抓好以对缅开放为重点的沿边开发开放，实现“五通”新突破。积极推动境外高速公路、铁路、滚弄大桥及清水河口岸基础设施建设，加快沿边高等级公路建设。争取中缅跨境运输车辆牌证互认。争取中方金融机构到缅方边境区域开设机构或网点，争取外汇特许兑换业务扩大到边境贸易政策落地临沧，积极推进跨国企业开展本外币资金集中运作。抓好清水河进境粮食指定口岸管理，加快推进进境食用水生动物、水果、植物种苗指定口岸申报工作，争取将临沧列为跨境动物疫病区域化管理及产业发展试点。深化中缅国际产能合作，鼓励境内企业到缅甸创业，吸引缅甸企业入临投资。争取创建临沧耿马（孟定）边境旅游试验区，提升已开通的三条边境旅游线路的运营成效。加快边民互市市场信息化建设及规范化管理，进一步做大边民互市。大力发展跨境电子商务。推动边境小额贸易向一般贸易、加工贸易、转口贸易发展，培育外贸竞争新优势。办好第七届边境经济贸易交易会。加强外事、侨务工作，扩大对缅文化、教育、卫生、青年、媒体、宗教等人文领域交流。

　　用足用好国家、省支持沿边重点地区开发开放政策，探索完善边合区管理体制机制，加快做大边合区经济规模。加快清水河口岸经济区规划建设。完成孟定清水河口岸改造工程和清水河、芒卡查验货场规范化建设，加快推进孟定清水河口岸迁址新建。加快推进亚行贷款项目等重大项目前期工作。加快发展商贸物流、进出口加工、装备制造、新材料等产业，逐步形成外向型产业集群。实现边合区规模以上固定资产投资增长20%以上，核心区招商引资实际到位资金、规模以上工业增加值增长10%以上。

　　深化与京津冀、泛珠三角、长三角、川渝等区域经贸合作。创新招商方式，规范招商引资流程，突出产业招商、园区招商，确保完成招商引资市外实际到位资金1161亿元。建立招商项目回访制，实行市级领导挂钩重点招商项目、招商引资专项督查督办等工作制度，为外来企业营造良好的投资发展环境。

　　（六）努力提升城乡人居环境，加快推进特色城镇化

　　坚持高起点规划、高标准建设、高水平管理，全面抓好孟定新城和临沧中心城建设。开工孟定综合交通枢纽示范城市建设项目，强化“门户型”、“枢纽型”城市功能，实现多种运输方式便捷换乘，努力把孟定建设成为中缅边境线上独具特色的新兴口岸城市。发挥临沧中心城对经济社会发展的引领和辐射带动作用，扩大城市规模，推动产业、人口聚集，努力建设区域性空港、铁路、公路交通枢纽和物流体系。

　　全面实施城乡“四治三改一拆一增”，建立责任街道包保制度，突出抓好拆除违规建筑和临时建筑，增加城镇绿化、水体、水果、花卉，坚决取缔占道经营，打通城市“断头路”“丁字路”。加大住房公积金投放，促进房地产市场健康发展。完成城镇棚户区改造4242户，加快城镇保障性住房、地下综合管廊、海绵城市、智慧城市建设，完成市级城市规划馆建设，开工市委党校搬迁项目，启动亚微展示中心建设。抓好耿马国家级中小城市综合改革试点。继续深化户籍制度改革，推进就地就近城镇化，力争常住人口城镇化率达40%。实施农村“七改三清”，启动一批特色小城镇建设，切实抓好71个省级示范村建设，加强农村坝区整治，实施旧村改造1333个，建设美丽宜居乡村400个，创建洁净村庄1200个，创建洁净庭院示范家园1.5万户。

　　（七）持续发展绿色循环经济，把生态优势转化成发展优势

　　持续推进“森林临沧”建设，加快创建国家森林城市。严守生态保护红线，完善生态补偿机制，加强国家公园、自然保护区、森林公园等建设与管理，严格保护生态区位重要和生态脆弱区，加强生物多样性保护。认真实施《临沧市古茶树保护条例》，推进古茶树资源的可持续利用。大力开展全民植树活动，完成新一轮退耕还林30万亩、陡坡地生态治理2万亩、新造林60万亩。加强地质灾害综合防治，完成水土流失治理290平方公里。

　　加快澜沧江绿色发展经济带和南汀河生物产业经济带建设，配套建设一批基础设施项目。扎实推进临沧国家可持续发展实验区和全国低碳国土实验区建设，积极创建国家级凤庆、沧源有机产品认证示范区，提升绿色发展水平。加快发展生态旅游、健康服务等绿色生态大健康产业。进一步巩固集体林权制度主体改革成果，加大林业龙头企业培育，提高林下经济产业化水平。发展绿色改造和节能环保产业，倡导和培育绿色生活方式，共享共建绿色家园。

　　实行最严格的环境保护。落实生态文明绩效考核、终身责任追究、生态审计制度，全面加强环境污染防治。继续推行“河长制”，加强对南汀河等主要流域的生态保护治理。加强集中式饮用水源地水质监测。推进煤炭行业和非煤矿山转型升级，加强污染源综合防控，提升城镇空气环境质量。整治农业面源污染。严格落实污染减排目标责任制，实施工业、建筑等重点领域能效提升计划，持续削减主要污染物排放总量。

　　（八）着力保障和改善民生，促进民族团结进步边疆繁荣稳定

　　深入贯彻党的民族宗教方针政策，维护和巩固民族宗教和谐稳定。启动全国民族团结进步示范市创建工作，实施好第二轮“一县十乡百村千户示范点创建工程”三年行动计划，抓好“直过民族”和人口较少民族精准脱贫，决不让一个兄弟民族掉队。实施少数民族传统文化抢救保护和世居少数民族文化精品工程，注重培养非物质文化遗产传承人，促进少数民族文化产业与生态文化旅游产业融合发展。

　　深入实施高校毕业生就业创业促进计划，加大创业担保贷款支持力度，完成城镇新增就业2.5万人。完成职业技能培训4.5万人、农村劳动力转移就业培训19万人。抓好城镇居民医疗保险和新农合的整合，建立统一的城乡居民医疗保险制度。新农合参合率保持在95%以上。完成各项社会保险参保401.3万人次。抓好被征地农民社会保障政策改革试点。

　　深化教育领域综合改革，扩大学前教育和高中阶段教育规模，学前三年毛入园率达65%以上，高中阶段毛入学率达80.5%以上，办好特殊教育，发展高等教育、继续教育、民办教育、职业教育，全面提高办学质量。推进义务教育均衡发展，义务教育巩固率达93%以上，加快实施“全面改薄”工程，边境县继续实行14年免费教育。加快创新型临沧建设，力争科技进步对经济增长的贡献率达47.5%。深化县级公立医院综合改革，推进医保支付方式改革，力争县域内就诊率提高到90%左右。发展中医药事业，完善疾病预防控制体系，做好妇幼保健和计划生育工作，继续实施全面两孩政策。加强文化阵地建设，开展知恩感恩教育。培育文化产业，发展文化事业。认真做好第十五届省运会、第十一届省少数民族传统体育运动会和第十一届省残疾人运动会筹备工作。组织好亚洲微电影艺术节等活动。全面放开养老服务市场，抓好社会救助兜底，支持残疾人事业发展。继续支持工会、共青团、妇联、科协、文联、工商联、社科联、关工委、妇儿工委做好工作。支持老龄、红十字、慈善等事业健康发展。进一步加强统计调查、测绘、粮食、参事、档案、地方志、人防、气象、水文等工作。

　　深入推进依法治市，抓好“七五”普法工作。切实做好人民调解工作，加快推进信访法治化，预防和化解社会矛盾。深入推进第四轮禁毒防艾人民战争。深化社会治安综合治理，严密防范和依法打击各种违法犯罪活动，提高群众安全感和满意度。支持驻临军警部队现代化建设、国防动员和民兵预备役工作。发挥党政军警民“五位一体”联动机制，持续推进“2·09”维稳后续工作，加快“智慧边境”建设，维护边境和谐安宁。推行“五个一”工作法，提高防灾减灾应急处置能力。加强食品药品质量安全监管，落实“四个最严”要求，保障群众“舌尖”上的安全。狠抓安全生产责任落实，强化重点行业领域专项整治，确保安全生产形势持续稳定。

　　继续办好10件惠民实事。

　　三、加强政府自身建设，全面提升政府的服务能力

　　强烈树立“不进则退、慢进也退”的理念，强化责任意识、忧危意识，大力弘扬“头顶理想、脚踩泥巴、心中有民、手上有招、团结干、创新干、拼命干”的临沧作风，全面加强政府自身建设，着力打造“临沧效率”，为实现“临沧速度”助推跨越式发展提供坚强保障。

　　严格依法行政。深化行政执法体制改革，强化规范、公正、文明和必要的综合执法，全面落实执法责任制，严格执行重大决定法制审核制度，科学决策，民主决策，建立健全责任追究制度。全面推行政府法律顾问制度。全力推进政府权力清单、责任清单、企业负面清单和信访诉求清单等制度实施，规范权力运行。重视发挥智库作用。遵循法治思维，做到法无授权不可为、法定职责必须为。

　　优化发展环境。不断深化“放管服”改革，巩固行政审批制度改革成果，实现“双随机、一公开”监管全覆盖，权责清单及时公开，全面推进政务公开和公共资源交易管理，大力发展电子政务，努力打造便捷高效的“互联网+政务服务”新模式。营造开放进取的思想环境，亲商、惠商的服务环境，诚信、守信的信用环境，平等、公正的法制环境，公开、规范的行政环境，文明、宽松的人文环境。

　　落实依法治政。坚持从严治党，强化主体责任，履行党风廉政建设“一岗双责”，继续推进惩治和预防腐败体系建设。认真落实中央八项规定，坚持不懈纠正“四风”，坚持勤俭办事，严格控制“三公”经费。加强行政监察，严格监管公共资金、公共资源、国有资产。建立健全行政绩效管理制度，实施工作目标倒逼管理，强化一线工作法，推进“马上就办”的效率机制建设。坚持正向激励，建立容错机制，对想干事、敢干事、干成事的干部给予褒奖，切实做到为敢于担当的干部担当，为敢于负责的干部负责。强化监督问责，对工作不力的干部约谈诫勉，对为官不为、懒政怠政的干部曝光、追责。自觉接受党内监督、人大监督、政协监督、审计监督、司法监督、社会监督和舆论监督，提高人大代表建议和政协提案办理的质量和水平，认真听取社会各界的意见建议，充分体现人民群众的意愿。

　　各位代表！让我们更加紧密地团结在以习近平同志为核心的党中央周围，在省委、省政府和市委的坚强领导下，以“党的光辉照边疆，边疆人民心向党”为主题，撸起袖子加油干、拼命干，努力完成今年经济社会发展目标任务，奋力推进临沧经济社会跨越式发展，以优异成绩迎接党的十九大胜利召开！
